# Wild and Hatchery Populations of Korean Starry Flounder (*Platichthys stellatus*) Compared Using Microsatellite DNA Markers

**DOI:** 10.3390/ijms12129189

**Published:** 2011-12-09

**Authors:** Hye Suck An, Soon Gyu Byun, Yi Cheong Kim, Jang Wook Lee, Jeong-In Myeong

**Affiliations:** Genetics and Breeding Research Center, National Fisheries Research and Development Institute, Namseoro 81-9, Gyeongsangnamdo 656-842, Korea; E-Mails: sgbyun@nfrdi.go.kr (S.G.B.); yckim@nfrdi.go.kr (Y.C.K.); lee9952@nfrdi.go.kr (J.W.L.); cosmo@nfrdi.go.kr (J.-I.M.)

**Keywords:** Korean starry flounder, *Platichthys stellatus*, microsatellite, genetic marker, genetic differentiation, heterozygosity

## Abstract

Starry flounder (*Platichthys stellatus*) is an important sport and food fish found around the margins of the North Pacific. Aquaculture production of this species in Korea has increased because of its commercial value. Microsatellite DNA markers are a useful DNA-based tool for monitoring the genetic variation of starry flounder populations. In this study, 12 polymorphic microsatellite DNA markers were identified from a partial genomic starry flounder DNA library enriched in CA repeats, and used to compare allelic variation between wild and hatchery starry flounder populations in Korea. All loci were readily amplified and demonstrated high allelic diversity, with the number of alleles ranging from 6 to 18 in the wild population and from 2 to 12 in the farmed population. A total of 136 alleles were detected at the 12 microsatellite loci in the two populations. The mean observed and expected heterozygosities were 0.62 and 0.68, respectively, in the hatchery samples and 0.67 and 0.75, respectively, in the wild samples. These results indicate lower genetic variability in the hatchery population as compared to the wild population. Significant shifts in allelic frequencies were detected at eight loci, which resulted in a small but significant genetic differences between the wild and hatchery populations (*F*_ST_ = 0.043, *P* < 0.05). Further studies with additional starry flounder sample collections are needed for comprehensive determinations of the genetic varieties between the wild and hatchery populations. These microsatellite loci may be valuable for future population genetic studies, monitoring the genetic variation for successful aquaculture management and the preservation of aquatic biodiversity.

## 1. Introduction

The Korean starry flounder *Platichthys stellatus* (Palas 1788) belongs to the family Pleuronectidae. This pleuronectid flatfish is distributed in countries surrounding the Pacific Ocean, stretching from the northeastern coast of Korea to Japan’s Sea of Okhotsk and from the Chukchi Sea, Bering Sea, and Aleutian Islands south to Los Angeles Harbor, California, USA [1]. This species appears to prefer shallow water (less than 73 m), however it has been recorded as deep as 274 m; young fish are often intertidal [2]. In Korea, *P. stellatus* is an important fishery resource that has been considered a target for prospective aquaculture diversification using production techniques similar to those previously developed for closely related pleuronectids, including *Paralichthys olivaceus*. Therefore, interest has been directed toward resource enhancement. Complete culturing including reproduction control and captive spawning, hatching, and larval and juvenile rearing are possible and, recently, artificially hatched juveniles of *P. stellatus* were released into Korean coastal breeding grounds for a sustainable fishery [3,4].

Although mass release of *P. stellatus* juveniles reared in hatcheries is expected to have an immediate effect on stock abundance, it could also cause changes in the genetic structure of wild populations. The reduced genetic diversity observed in most hatchery stocks may have detrimental effects on commercial traits such as growth rate, survival and disease resistance, which can post a great risk for aquaculture [5,6]. Thus genetic monitoring for hatchery stocks and natural populations is recommended to preserve genetic variation in natural populations [7]. Therefore, it is vital and critical to investigate the genetic variability of wild and cultured starry flounders for the management of wild populations and successful aquaculture.

Molecular markers are an important tool for evaluating levels and patterns of genetic diversity and have been used to study genetic diversity in a number of fish species [8]. Among various molecular markers now available to study genetic diversity in different fish species, microsatellites (MS) are markers of choice because of their highly polymorphism with codominant inheritance [9,10]. Microsatellites have been used to monitor genetic differences between hatchery stocks and wild populations in various fish species [11–13]. However, despite the importance of starry flounder for commercial aquaculture in Korea, there are only a limited number of MS markers available [14]. Furthermore, no study has focused on the genetic variability and population structure of this species. Therefore, additional highly informative microsatellite markers need to be developed and screened to identify markers that are the most informative for various other applications, including studies of genome mapping, parentage, kinships and stock structure. The present study is aimed at identifying new microsatellite loci and comparing the genetic similarity and differences of wild and hatchery starry flounder populations in Korea.

## 2. Results and Discussion

### 2.1. Microsatellite Marker Isolation

In total, more than 500 white colonies were obtained from the transformation with the Korean (CA)*_n_*-enriched genomic DNA library, approximately 200 of which were screened by PCR for the presence of a repeat-containing insert. Sequencing of the inserts from these 200 colonies revealed 130 loci containing MS arrays with a minimum of five repeats, corresponding to an enrichment efficiency of 26%. These were primarily 2-bp repeat motifs, some of which were combined with other 2-bp repeat motifs. Primers were designed and tested for 53 loci that exhibited adequately long (>20 bp) and unique sequence regions flanking the MS array. Seventy-seven loci were discarded because the MS sequences were so close to the linker sequence that primer sequences could not be designed for amplification. After initial PCR assays, only 17 primer sets (KPs1, KPs2, KPs3A, KPs5A, KPs12B, KPs15, KPs17A, KPs18, KPs20, KPs23, KPs25, KPs27, KPs29, KPs31, KPs32, KPs33, and KPs36) successfully yielded variable profiles. The remaining 36 primer sets gave either inconsistent or no PCR products, despite adjusting the dNTP concentrations and using an annealing temperature gradient. An initial evaluation of the polymorphic status of each locus was done by genotyping in 16 individuals randomly selected from the wild population. All loci were polymorphic with the exception of KPs2, KPs5A, KPs 23, KPs29, and KPs31, which had one allele, showed great allele varieties with clear peak patterns and thus suited for further investigation. The primer sequences, repeat motifs, annealing temperatures, fluorescent labels, and GenBank accession numbers for the 17 newly identified MS loci are summarized in Table 1. A homology search using BLAST program showed that none of these 17 sequences had similarity to any of the sequences in the GenBank.

Generally, in case of magnetic bead-based enrichment, the types and ratios of biotin-labeled probes and the positive clone selection strategy can affect the success of cloning and the efficiency of enrichment. In this study, we created MS libraries enriched for CA repeat sequences by following the protocol of Hamilton *et al*. [15] with modifications that have been previously described [16,17]. Of the positive clones obtained, about 26% contained microsatellite repeats (130 of 500); this number is comparable with number obtained from the normalized cDNA library enriched for CA-repeats for Asian seabass, *Lates calcarifer* (26.5%) [18], but lower than that for flounder (74%) [19] and tilapia (96%) [17]. Except for the efficiency of enrichment procedure, the differences in enrichment efficiency are probably a result of the use of different biotin-labeled oligonucleotide probes and the proper ratio. In the case of tilapia, a variation of the hybrid capture method was used, which is likely a reflection of the relative complexity of several enriched libraries with different size selection of the restricted genomic DNA. In the genome of bivalves, however, remarkable differences in microsatellite density among closely related species were suggested [20].

### 2.2. Genetic Variation within Populations

Samples of 48 wild and 30 hatchery-bred *P. stellatus* collected from around the eastern coast of Korea were screened for variation at the 12 new polymorphic MS loci. The 12 primer sets yielded variable profiles; reruns were conducted for 20% of the samples to ensure that the allele scoring was reproducible. No differences were observed, indicating that there were no genotyping errors. Samples that failed to amplify after the rerun were not included, which made it unlikely that poor DNA quality affected our results.

The MICRO-CHECKER analysis showed that some loci may have been influenced by one or more null alleles in both the wild and hatchery samples; our data showed that loci KPs1, KPs17A, and KPs32 in the farmed samples and loci KPs1, KPs17A, and KPs36 in the wild population were affected. Loci KPs1 and KPs17A appeared to be influenced in both the wild and hatchery samples, indicating that using loci KPs1 and KPs17A for population genetic analyses that assume Hardy-Weinberg equilibrium (HWE) may prove to be problematic. However, loci KPs32 and KPs36 were affected by null alleles in only one sample; thus, they were included in further analyses.

All 12 MS loci were found to be highly polymorphic in both populations. A total of 136 different alleles were observed and the average number of alleles per locus was 11.3. The number of alleles varied from two at the KPs18 and KPs20 loci to 18 at the KPs12B locus (Table 2). Not all loci were equally variable. Especially, KPs12B, KPs17A, KPs25 and KPs27 displayed greater allelic diversity, as well as higher levels of heterozygosity. The observed heterozygosity ranged from 0.24 at locus KPs18 to 0.94 at KPs27, whereas expected heterozygosity varied from 0.20 at locus KPs18 to 0.89 at KPs27 (Table 2). Due to the difference in sample size of the wild and hatchery populations, the parameter allelic richness (*A*_R_) was employed to compare different populations independent of sample size. Overall allelic richness varied from 2 to 14.81 (Table 2).

In this study, a high level of genetic diversity (mean heterozygosity = 0.75; mean allelic number (*N*_A_) = 10.75) in the wild population was detected, although *N*_A_ is dramatically lower than the reported *N*_A_ for marine fish (*N*_A_ = 19.9 ± 6.6 averaged from 12 species) [21]. However, the mean observed (*H*o = 0.67) and expected heterozygosity (*H*e = 0.75) of starry flounder was comparable to other marine fish species (H = 0.77 ± 0.19 averaged from 12 species) [13,21], suggesting that these polymorphic microsatellites may be sufficient to reveal intraspecific diversity of *P. stellatus*.

Inbreeding coefficients (*F*_IS_) varied among markers from −0.06 (KPs27) to 0.41 (KPs17A) in the hatchery samples, and from −0.18 (KPs3A) to 0.66 (KPs17A) in the wild samples. Average *F*_IS_, including all markers, was 0.11 in the hatchery samples and 0.06 in the wild samples (Table 2).

**Figure 1:**
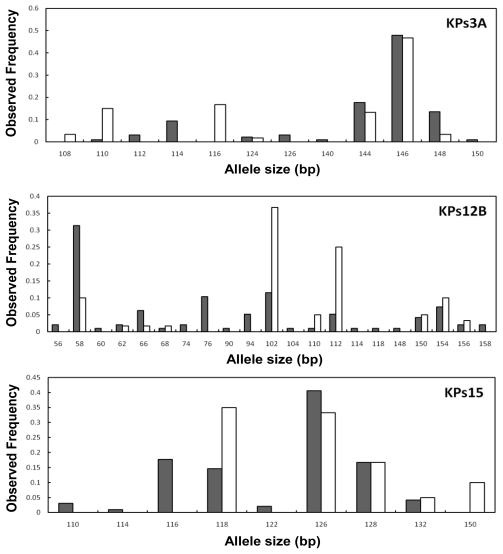

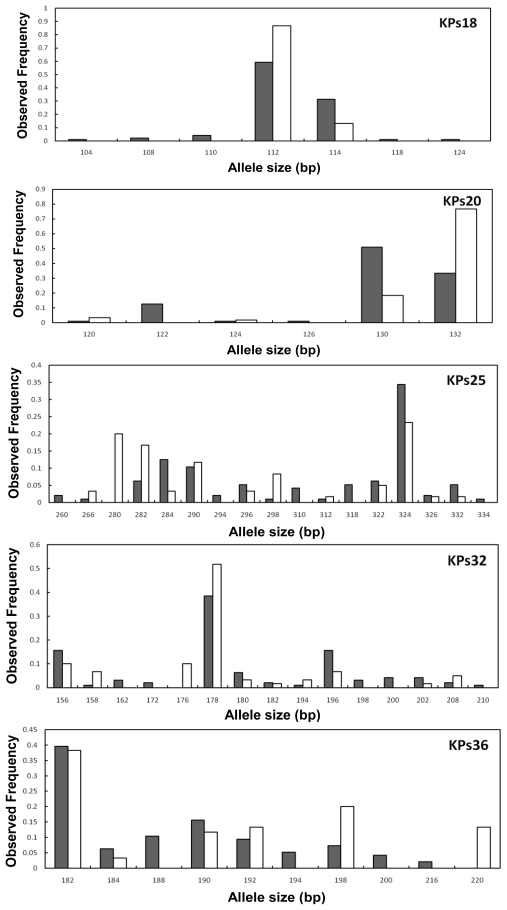
Allele frequency distributions at the eight microsatellite loci which showed significant heterogeneity from the wild (closed box) and hatchery (open box) populations of *Platichthys stellatus* used in this study.

There was significant heterogeneity between wild and hatchery allele frequencies for eight loci following sequential Bonferroni correction for multiple tests (*P* < 0.004) (Table 3, Figure 1). The maximum number of alleles was detected at locus KPs12B (*n* = 18), and the allele frequencies at this locus were clearly different between the wild and hatchery populations. Distinct differences in the allele frequencies between the wild and hatchery populations were also observed at the loci, KPs3, KPs15, KPs18, KPs20, KPs25, KPs32, and KPs36. Common alleles shared across wild and hatchery populations. More unique alleles were observed in the wild population (49) than the hatchery population (7), though their frequencies were very low (Table 2). Allele frequency distributions indicated the presence 32 rare alleles (frequency < 5%) of a total of 87 alleles over all loci (mean 36.8%) in the farmed sample, whereas 68 rare alleles of a total of 129 alleles (mean 52.7%) were observed in the wild sample (data not shown). Rare alleles were detected at most loci and were never associated with a particular locus in either population. No significant linkage disequilibrium between all pairs of the 12 microsatellite loci was detected (*P* > 0.004).

Significant departures from HWE, even after Bonferroni correction (*P* < 0.004), were found at three loci (KPs1, KPs17A, and KPs32) in the hatchery samples and three loci (KPs1, KPs17A, and KPs36) in the wild samples, all due to heterozygote deficiency.

In the hatchery populations, homozygote excess is commonly caused by a limited number of founders or founder effects. In the wild populations, homozygote excess could be explained by a population effect such as the Wahlund effect or inbreeding or the effective population size and artificial and natural selection during seed production and cultivation [22]. However, these explanations seem unlikely because other loci were consistent with HWE expectations. Thus, a likely explanation for the observed heterozygote deficit in our microsatellite data might be primer site sequence variation resulting in null alleles. Null alleles, a locus-dependent effect found frequently at microsatellite DNA loci, are the most likely cause of the heterozygote deficiency in HWE tests [23]. A high frequency of null alleles complicates many types of population genetic analyses that rely on HWE because false homozygotes are common [24]. Indeed, our MICRO-CHECKER analysis revealed the presence of null alleles at those loci, with a significant heterozygote deficit. Therefore, most deviations from HWE in this study might have been due to the presence of null alleles resulting from base substitutions or deletions at the PCR priming sites in the flanking region of the microsatellites. The importance of null alleles as an explanation for heterozygote deficiency has been reported for other marine fish [19,25].

Considering that this study was limited by the number of populations screened, the genetic diversity parameters for each population may be explained by data from additional populations, which may allow for a more precise genetic characterization of the MS loci used. Therefore, our results should be interpreted with caution. Further study is required to assess the genetic resources of wild populations and the influence of aquaculture on the genetic structure of this important fishery species.

### 2.3. Genetic Differentiation between the Wild and Hatchery Populations

The wild population had a higher number of alleles and a higher allelic richness than the hatchery-bred population. A statistically significant (*P* < 0.05) reduction of allele richness was observed in the hatchery-bred individuals of starry flounder (mean = 7.25) compared to the wild population (mean = 9.50). The mean observed and expected heterozygosities were 0.667 and 0.749 respectively in the wild samples and 0.620 and 0.677 respectively in the hatchery samples. However, genetic diversity in terms of heterozygosity was not markedly reduced (*P* > 0.05) in contrast to the significant reduction of allelic richness. Loss of rare alleles in population can greatly influence allelic richness, but has little effect on heterozygosity [26]. This non significant reduction in heterozygosity in cultured stock in contrast to wild population was reported in other studies [27].

*F*_ST_ estimates were significantly different between the hatchery and wild populations whether or not the two loci with potential null alleles (KPs1 and KPs17A) were included (*F*_ST_ = 0.043, *P* < 0.01 and *F*_ST_ = 0.053, *P* < 0.01, respectively) (Table 2). The significant *F*_ST_ estimates indicate the presence of genetic differentiation between the populations, which was likely a result of reduced genetic variation. For starry flounder in Uljin, Korea, the progeny produced for release had a different genetic composition with significant reductions in genetic diversity compared with the wild population, although with no significant reductions in mean heterozygosity (*P* > 0.05; Table 2). In fact, the loss of alleles is more important than a change in allele frequencies because the latter can be changed again by random drift; no way exists to recover a lost allele. The decline of genetic variation in cultured stock may be caused by the increased effect of genetic drift resulting from using a small number of parental individuals and artificial selection existing in the hatchery environment [28]. Reduced genetic variation can result in reduced performance in aquaculture because this is the source of variation for important traits such as growth rate and disease resistance [5,29]. For proper management of stock enhancement programs, monitoring for genetic structure and diversity must be considered in addition to biological, ecological, and fishery factors. Therefore, samples from the wild population should be taken and analyzed with genetic markers before the fish is used as broodstock. A sample of hatchery-reared fish should then be taken for genetic analysis. This information will be useful in evaluating the feasibility of the enhancement program to maintain the genetic diversity of wild populations, as well as to improve hatchery management for the production of high quality starry flounder.

## 3. Experimental Section

### 3.1. Sample Collection and DNA Extraction

For genomic DNA isolation of high-molecular-weight and microsatellite enriched partial genomic library construction, fin clips were collected from an individual starry flounder from Uljin, Korea. Samples of 48 wild and 30 hatchery-bred starry flounders were collected at the East Sea Fisheries Research Institute of the National Fisheries Research and Development Institute in Uljin, Korea in 2006. Wild starry flounders were sampled from broodstock that was captured from the eastern coast of Korea since 2000 and farmed samples were obtained from the first generation of hatchery-reared stock produced in 2004. In general, both wild caught and captive cultured sources of the broodstock were used for artificial reproduction. However, details of the exact proportion of these two different sources of individuals and their origins on the cultured stock were not available. All the samples were placed in absolute ethanol and kept frozen at −20 °C until DNA extraction. The TNES-urea buffer method [30] was used to isolate high-molecular-weight DNA for microsatellite isolation. For genotyping, total DNA from fin-clips of each sample was extracted using a MagExtractor-Genomic DNA Purification Kit (TOYOBO, Osaka, Japan) for an automated DNA extraction system, MagExtractor MFX-2100 (TOYOBO, Osaka, Japan). Extracted genomic DNA (20 μg) was stored at −20 °C until further use for PCR.

### 3.2. Microsatellite-Enriched Genomic Library Construction and Microsatellite Sequencing

A partial genomic library enriched for CA repeats was constructed using a slightly modified enrichment procedure with pre-hybridization polymerase chain reaction (PCR) amplification, as described previously [16,17]. The extracted DNA was digested with the restriction enzymes *Alu*I, *Rsa*I, *Nhe*I, and *Hha*I (New England Biolabs, Beverly, MA, USA). DNA fragments in the range of 300–800 bp were isolated and purified using the QIAquick Gel Extraction Kit (Qiagen, Hilden, Germany). The selected fragments were ligated to an adaptor (SNX/SNX rev linker sequences), and the linker-ligated DNA was amplified using SNX as a linker-specific primer for PCR. For enrichment, the DNA was denatured and biotin-labeled repeat sequences ((CA)_12_GCTTGA) [31] were hybridized to the PCR products. The hybridized complex was separated with streptavidin-coated magnetic spheres (Promega, Madison, WI, USA). After washing, the bound, enriched DNA was eluted from the magnetic spheres and re-amplified with an adaptor sequence primer. PCR products were purified using a QIAquick PCR Purification Kit (Qiagen, Hilden, Germany).

### 3.3. Isolation of Microsatellite-Containing DNA Fragments and Microsatellite Sequencing

The purified PCR products were digested with NheI, cloned using an XbaI-digested pUC18 vector (Pharmacia, Piscataway, NJ, USA), and transformed into *Escherichia coli* DH5α competent cells. White colonies were screened for the presence of a repeat insert by PCR using the universal M13 primer and non-biotin-labeled dinucleotide primers. PCR products were examined on 2% agarose gels, and inserts producing two or more bands were considered to contain a microsatellite locus. Positive clones were cultured and purified. Plasmids from insert-containing colonies were recovered using the QIAprep Spin Miniprep Kit (Qiagen, Hilden, Germany) and sequenced using the BigDye Terminator Cycle Sequencing Ready Reaction Kit (ver. 3.1; Applied Biosystems, Foster City, CA, USA) and an automated sequencer (ABI Prism 310 Genetic Analyzer, Applied Biosystems, Foster City, CA, USA).

### 3.4. Primer Design and Allele Scoring

Primers were designed based on sequences flanking the MS motifs using the OLIGO software package (ver. 5.0; National Biosciences, Plymouth, MN, USA). A gradient PCR was performed on each primer pair to optimize the annealing temperature (ranging from 50–60 *°*C) using eight starry flounders captured from Uljin, Geongsangbukdo, Korea. The PCR amplification was performed using a PTC 200 DNA Engine (MJ Research, Ramsey, MN, USA) in a 10-μL reaction containing 0.25 U of *Ex Taq* DNA polymerase (TaKaRa Biomedical, Shiga, Japan), 1 × PCR buffer, 0.2 mM dNTP mix, 100 ng of template DNA, and 10 pmol of each primer, where all forward primer were labeled with 6-FAM, NED, and HEX dyes (Applied Biosystems, Foster City, CA, USA). The PCR reaction ran for 11 min at 95 *°*C, followed by 35 cycles of 1 min at 94 *°*C, 1 min at the annealing temperature (Table 1), and 1 min at 72 *°*C, with a 5-min final extension at 72 *°*C. Microsatellite polymorphisms were screened using an ABI PRISM 3100 Automated DNA Sequencer (Applied Biosystems, Foster City, CA, USA) and alleles were designated by PCR product size relative to a molecular size marker (GENESCAN 400 HD [ROX], Applied Biosystems, Foster City, CA, USA). Fluorescent DNA fragments were analyzed using the GENESCAN (ver. 3.7) and GENOTYPER (ver. 3.7) software packages (Applied Biosystems, Foster City, CA, USA).

### 3.5. Sample Comparisons

Samples were screened for variation at the newly developed MS loci. MICRO-CHECKER 2.2.3 [32] was used to detect genotyping errors due to null alleles, stuttering, or allele dropout using 1000 randomizations. For genetic diversity parameters, the number of alleles per locus (*N*_A_), size of alleles in base pairs (*S*), frequency of the most common allele (*F*), and number of unique alleles (*U*) were determined for each local sample at each locus using the program GENEPOP (version 4.0) [33]. This was also used to identify deviation from Hardy-Weinberg equilibrium (HWE; exact tests, 1000 iterations) and the observed and expected heterozygosities, indicating an excess or deficiency of heterozygotes. FSTAT (version 2.9.3.2) [34] was used to calculate the inbreeding coefficient (*F*_IS_) [35] per locus and sample and allelic richness (*A*_R_) [36], suitable for comparing the mean number of alleles among populations regardless of sample size. ARLEQUIN was used to assess linkage disequilibrium for all pairs of loci, whose empirical distribution is obtained by a permutation procedure [37] and to calculate single-locus and global multilocus values (*F*_ST_; 1000 permutations) [35]. Significance levels were adjusted for multiple tests by using sequential Bonferroni correction [38]. The significance for differences in the genetic diversity of both samples was tested using Wilcoxon sign rank test.

## 4. Conclusions

In conclusion, genetic studies on starry flounder with microsatellite DNA markers are very rare with only one recent report on the development of microsatellites as a tool for discriminating a hybrid between olive flounder and starry flounder [14]. No detailed information is available to date on the genetic diversity of wild and cultured stocks of starry flounder. In this study, microsatellite-enriched genomic library of starry flounder was constructed and a total of 12 highly polymorphic microsatellite loci were characterized and used to study genetic differences between the wild and hatchery populations. This study demonstrated that genetic changes, including reduced genetic diversity and significant differentiation, have taken place in hatchery starry flounder stock compared to the wild population due to random genetic drift during aquaculture practices. For starry flounder in Korea, genetic variation changes of the cultured stock relative to wild population should not be neglected in the stock enhancement program. Continued monitoring of genetic variance with additional starry flounder sample collections in broader perspective is essential for the establishment of suitable guidelines for resource management and selective breeding. The use of these novel microsatellite markers will certainly facilitate this purpose.

## Figures and Tables

**Table 1:** Characteristics of the 17 microsatellite loci isolated from *Platichthys stellatus*.

Locus	Repeat motif	Primer sequence (5′ → 3′)	*T*a (°C)	*Genbank accession no.*
KPs1	(CA)_11_	CAGCAGTAAGAGTGTGTCCTG **Hex** TTCAGCCTGTTTTCTGTCAT	55	EF157643
KPs2	(TG)_12_	TTAGGGGTGGGACAGACT **Hex** GTCATCAGATGGGAGAAAGAT	55	EF157644
KPs3A	(TG)_12_	AGGTTATCGCATCTGTGTGAT **Hex** GAACTCTTGTTTCGCTTCAG	55	EF157645
KPs5A	(CA)_11_	ACAGATAGCAAGGTCATAGAA **Ned** TACTAATTCCAAGGTGTTGAT	60	EF157646
KPs12B	(TG)_15_TC(TG)_12_GT(TG)_3_	TGTTTACTGCTTTCCTGTGTG **Fam** TGTATTTCAGCCTGCTTTATG	58	EF157647
KPs15	(TG)_5_TA(TG)_8_TC(TG)_4_	GAGCCAGACCTCTCATGTTAC **Fam** CGCTTCCATGTGAACCAG	60	EF157648
KPs17A	(TG)_5_TC(TG)_9_	CAACCACGTTATCCTCTGTG **Fam** CCAGAATAAATCTCATGCTCA	60	EF157649
KPs18	(TG)_8_CG(TG)_4_CG(TG)_3_	TCTTGGATGTAGTGTATGTGC **Fam** GAAAACACGAAATTTGACAG	60	EF157650
KPs20	(CA)_14_	TGGGCAACTACGTACACACTA **Fam** GCCGACATTACAAAAACAAA	58	EF157651
KPs23	(CA)_9_CG(CA)_5_CG(CA)_20_TA(CA)_5_	ACAAACACTTGCATGGGTAT **Ned** ATCCTCTAGCAGAAGCATTTC	55	EF157652
KPs25	(TG)_6_TA(TG)_8_TA(TG)_10_	TGTTATCGGGTGTTGATTGTA **Ned** GTTGATTGTGAAACGCTGTT	60	EF157653
KPs27	(CA)_27_	TGTTGAAATCTAATGGGCTAT **Fam** GTGCGCAGCAAAAACTA	51	EF157654
KPs29	(CA)_6_AA(CA)_14_	TCCTACTCTACACACCCACAT **Hex** GGATCGGAAAAGACAGACTA	55	EF157655
KPs31	(CA)_10_	TGATTTCCAATTACTCACATA **Fam** CTAGCAGAAGCCTTTCAG	58	EF157656
KPs32	(TG)_8_TT(TG)_7_TA(TG)_22_	TTAAATAAGTGTCTGGGGATT **Hex** GCCACACTTCTGCTTCTG	55	EF157657
KPs33	(CA)_9_-(CA)_11_	TTTCACTTCTCTTTGGGTTAC **Ned** GGCAGACTGATTCCTCATT	60	EF157658
KPs36	(TG)_4_TT(TG)_13_	ATGTGCCCAATAAAACAAAC **Hex** CTAAGCCCTAGAGCAAACAGT	58	EF157659

*Ta* is the optimal annealing temperature.

**Table 2:** Summary of the statistics for 12 polymorphic microsatellite loci in the two *Platichthys stellatus* populations.

Population (No)		Microsatellite Loci												
		KPs1	KPs3A	KPs12B	KPs15	KPs17A	KPs18	KPs20	KPs25	KPs27	KPs32	KPs33	KPs36	Mean
	*F*_ST_	0.012	0.065	0.038	0.028	0.102	0.135	0.035	0.007	0.015	0.038	0.008	0.035	0.043
Uijin Captured (48)	*N*_A_	10	9	18	7	11	7	6	16	14	14	8	9	10.75
	*A*_R_	9.06	7.92	14.81	6.57	10.10	5.72	4.88	14.05	12.64	12.32	7.09	8.84	9.50
	*S*	140–174	110–150	56–158	110–132	62–104	104–124	120–132	260–334	82–130	156–210	274–294	182–216	
	*F*	0.385	0.531	0.313	0.563	0.208	0.594	0.479	0.344	0.208	0.385	0.510	0.396	0.41
	*U*	5	5	8	2	2	5	4	5	3	5	1	4	4.08
	*H*e	0.819	0.667	0.868	0.630	0.873	0.583	0.528	0.845	0.888	0.832	0.661	0.793	0.749
	*H*o	0.483	0.698	0.833	0.625	0.633	0.553	0.475	0.792	0.938	0.763	0.604	0.604	0.667
	*F_IS_*	0.297 (0.000)	−0.176 (0.147)	0.100 (0.486)	0.078 (0.661)	0.355 (0.000)	0.088 (0.453)	0.151 (0.014)	0.041 (0.399)	−0.117 (0.580)	0.097 (0.028)	0.050 (0.208)	0.211 (0.009)	0.061 (0.120)
	*P*	0.000	0.069	0.376	0.784	0.000	0.628	0.009	0.475	0.661	0.010	0.148	0.000	0.074
Uljin Cultured (30)	*N*_A_	5	7	10	5	9	2	2	12	12	10	7	6	7.25
	*A*_R_	5.00	7.00	10.00	5.00	9.00	2.00	2.00	12.00	12.00	10.00	7.00	6.00	7.25
	*S*	142–170	108–148	58–156	118–150	68–104	112–114	120–132	266–332	82–140	156–208	274–294	182–220	
	*F*	0.517	0.683	0.367	0.367	0.283	0.867	0.767	0.233	0.283	0.600	0.500	0.317	0.48
	*U*	0	3	0	0	0	0	0	1	1	1	0	1	0.58
	*H*e	0.683	0.512	0.867	0.800	0.868	0.200	0.364	0.871	0.867	0.627	0.666	0.800	0.677
	*H*o	0.533	0.578	0.789	0.743	0.667	0.235	0.300	0.865	0.897	0.467	0.633	0.733	0.620
	*F_IS_*	0.364 (0.016)	−0.031 (0.848)	0.080 (0.290)	0.007 (0.094)	0.405 (0.044)	−0.055 (0.414)	0.046 (0.025)	0.063 (0.312)	−0.056 (0.243)	0.299 (0.053)	0.087 (0.349)	0.100 (0.013)	0.109 (0.215)
	*P*	0.000	1.000	0.021	0.016	0.000	0.428	0.012	0.156	0.189	0.000	0.352	0.023	0.011

Single-locus *F*_ST_, number of samples (No), number of alleles per locus (*N*_A_), allelic richness (*A*_R_), size of alleles in bp (*S*), frequency of the most common allele (*F*), number of unique alleles (*U*), expected heterozygosity (*H*_e_), observed heterozygosity (*H*o), inbreeding coefficient (*F*_IS_), and probability of a significant deviation from Hardy-Weinberg equilibrium after the Bonferroni correction (*P*, initial α = 0.05/12 = 0.004) are given for each population and locus. Calculations assume that individuals with one microsatellite band are homozygous for the allele. Number in parenthesis below *F*_IS_ indicates the probability of significant heterozygosity excess or deficit.

**Table 3:** Comparison of allele frequencies between the wild and hatchery populations at 12 microsatellite loci of *Platichthys stellatus.*

Locus	*P*-value	Locus	*P*-value
KPs1	0.016	KPs20	0.000*
KPs3A	0.000*	KPs25	0.000*
KPs12B	0.000*	KPs27	0.577
KPs15	0.000*	KPs32	0.003*
KPs17A	0.461	KPs33	0.352
KPs18	0.002*	KPs36	0.000*

Probability values of homogeneity of allelic frequency distributions (*P*) estimated by a test analogous to the Fisher exact test in the Markov-chain method are shown; wide significance levels were applied using the sequential Bonferroni technique (*k* = 12), Significant at *P* < 0.004.
